# Supplementary material for: Towards accurate differential diagnosis with large language models
Source: Nature. 2025 Apr 9;642(8067):451–7. doi: 10.1038/s41586-025-08869-4 (PMC12158753; doi:10.1038/s41586-025-08869-4)
Supplement: Supplementary file 1 — Supplementary Figs. 1 and 2 and Supplementary Tables 1 and 2. [file 41586_2025_8869_MOESM1_ESM.pdf]

nature portfolio

https://doi.org/10.1038/s41586-025-08869-4

Supplementary information

Towards accurate differential diagnosis with large language models

In the format provided by the authors and unedited

Supplementary Information

SI.1 Contamination Analysis

We trained AMIE by building on an model pretrained on large-scale data and fine-tuning on medical data. While we did not include NEJM case reports in the fine-tuning data for the model, it is possible that pretraining data for the model contained partial mentions or full NEJM case reports, whether from the original source (NEJM) or reproduced by other websites. To better understand the possibility of overlap between the training corpus and the test data, we performed a contamination analysis. The fine-tuning data did not include any NEJM case report text; however, the pretraining corpora for the base LLM may have. We looked for overlap between character sequences in the test articles and training corpora using a sliding window, searching for all instances of at least 512-character overlap. A case report is considered to have overlap if at at least one document from the pretraining corpora has an overlap.

We identified that there was no overlap for case reports beginning in 2022 and 2023. Some overlap existed for case reports published prior to 2022. We calculated the top-N accuracy for AMIE on both of these sets of case reports, prior to 2022 (N = 238) and 2022 to date (N = 65), and did not observe a substantial difference in results. Across all years, 16.9% (51 out of 302) of case reports displayed at least one instance of overlap.

The DDx quality scores remained significantly higher for clinicians assisted by AMIE (top-10 accuracy 52.3%) compared to clinicians without its assistance (34.6%) (McNemar's Test: 15.2, $p < 0.01$) on the no-overlap set. The quality score for AMIE (55.4%) was also higher than that for the clinicians without assistance (McNemar's Test: 14.4, $p < 0.01$). However, the difference between clinicians after assistance in the Search (46.2%) and AMIE conditions (52.3%) was not significant (McNemar's Test: 0.73, $p = 0.39$). This final result may be a consequence of the reduced sample size (N=56).

We performed an additional overlap analysis excluding articles with greater than 512- characters overlap (N=53 articles excluded, N=249 articles retained). This represents a larger percentage of the cases some of which have some text overlap with the data in the training set, but only a small portion of the case text. Once again, we did not observe a large difference in results overall.

In this case all the differences that were significant on the full set remain so, the DDx quality score remained significantly higher for clinicians assisted by AMIE (top-10 accuracy 51.4%) compared to clinicians without its assistance (33.1%) (McNemar's Test: 38.6, $p < 0.01$) on the uncontaminated set. The quality score for AMIE (61.4%) was also higher than that for the clinicians without assistance (McNemar's Test: 38.6, $p < 0.01$) and the difference between clinicians after assistance in the search (44.2%) and AMIE conditions (52.4%) was significant (McNemar's Test: 3.95, $p = 0.05$).

SI.2 Model Consistency

LLMs can be non-deterministic if they have a temperature that is non-zero or if the batch size is changed. We generated 10 repetitions of the DDx lists from AMIE for all 302 cases with temperature=0.5 (Fig. R1(a)). We then performed auto evaluation on these using Med-PaLM 2. We also performed experiments with temperature settings of 0, 0.2, 0.4, 0.6, 0.8, 1.0 (Fig. R1(b)). These results show that the model produces consistent results. Better performance is generally obtained with a non-zero temperature. A temperature of 0.5 was used for all the experiments in the main article.

SI.3 Rater Consistency

Comparing the two conditions, the average scores for appropriateness and comprehensiveness of the DDx lists without assistance were slightly different; however, performing Wilcoxen signed-rank tests the appropriateness scores and comprehensiveness scores were not statistically different suggesting consistency among distinct groups of raters under the same unassisted circumstances.

Specifically, the Appropriateness scores were not significantly different in the Search (condition I) baseline

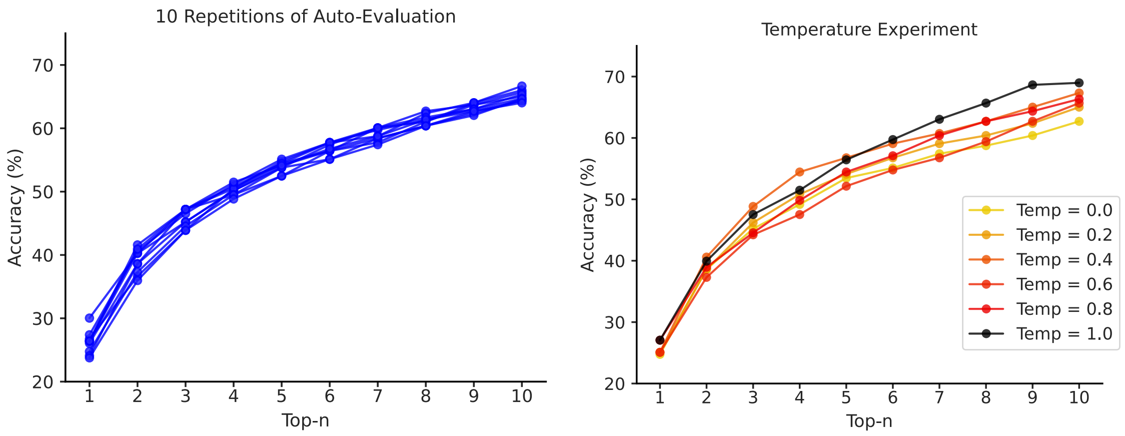

Figure SI.1 | Consistency of Top-n Accuracy. (left) AMIE top-n performance, based on auto-eval using Med-PaLM 2, repeated 10 times. Temperature=0.5. (right) AMIE top-n performance, based on auto-eval using Med-PaLM 2, with different temperature settings.

and AMIE (II) condition baseline (Search Baseline = 3.71, AMIE Baseline = 3.75, Wilcoxon signed-rank test $p = 0.63$).

The number of cases that scored 4 for Comprehensiveness (i.e., The DDx contains all candidates that are reasonable) was not statistically different for clinicians in the Search (condition I) baseline and AMIE (II) condition baseline (McNemar's Test: $p = 0.23$).

The quality scores were slightly different between the two baselines. The number of cases that scored 5 (i.e., The DDx included the top diagnosis) was higher in the Search condition baseline (33.8%) compared to the AMIE condition baseline (27.2%) (McNemar's Test: $p = 0.03$).

To further consolidate this point we ran a linear mixed effects models to test the effect of the Arm (either Assisted by Search=0 or Assisted by AMIE=1) on the final diagnosis score after assistance while controlling for the effect of baseline (unassisted final diagnosis score). The results were as follows:

Table SI.1 | Linear Mixed Effects Model Testing the Effect of the Study Arm. Mixed effects models to test the effect of the Arm (either Assisted by Search=0 or Assisted by AMIE=1) on the final diagnosis score after assistance while controlling for the effect of baseline (unassisted final diagnosis score).

	Coefficient	Std. Err.	z	P>|z|	CI: 0.025	CI: 0.975
Intercept	1.438	0.120	12.028	0.000	1.203	1.672
Arm	0.378	0.085	4.451	0.000	0.212	0.545
Baseline	0.620	0.030	20.338	0.000	0.560	0.680
Group Var	0.001					

SI.4 Including Laboratory Data Tables

The case texts used in our experiments represent a synthesis of available clinical data and therefore do routinely include a subset of key lab values (though not all lab values). We ran experiments in which we included the tabular lab data in the prompt to compare the performance with a model that does not have access to the lab tables. An example prompt would be:

Case: [Case Text]
Laboratory Data: [Lab Text Tab Delimited]

What are the top 10 most likely diagnoses? Be precise, listing one diagnosis per line, and try to cover many unique possibilities (at least 10). The top 10 diagnoses are:

For the 302 cases, 239 had specific tables of "Laboratory Data". We included the contents of these as tab delimited text in the query with the case text. We performed auto-eval on 20 repetitions of the DDx task with Temperature=0.5. The Top-N results (see Fig. R2 below) were not statistically different compared to the results without labs included in the input.

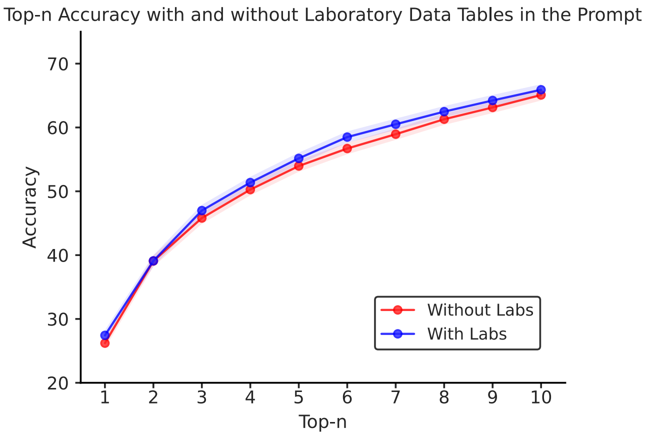

Figure SI.2 | Temperature Consistency of Top-n Accuracy. AMIE top-n performance based on auto-evaluation for different temperature settings.

SI.5 Qualitative Analysis

We used semi-structured qualitative interviews to gather information from participating clinicians on their experiences of using the tool, their views of the potential role and risks of LLMs in medical diagnosis and in aiding the differential diagnosis process. These interviews highlighted the potential for LLMs to increase the breadth of DDx lists and speed up the process of arriving at a comprehensive DDx for challenging cases. The clinicians also highlighted that the most appropriate application at the present time would be in learning and education.

We describe our qualitative results that provide insight into how the clinicians viewed AMIE. We identified several key themes in the clinicians' responses and present illustrative quotes below.*

LLM for Generating Comprehensive Differentials.

AMIE was particularly effective at broadening the scope of differential diagnoses, prompting clinicians to consider diagnoses they might not have initially considered, particularly in less familiar medical specialties (C3, C5). They noted that this was helpful when their initial differential was limited, or when they were uncertain about the diagnosis and struggled to identify useful search terms. This insight aligns with C2's observation that search tools were less helpful when they were unsure of the potential diagnoses.

One clinician contrasted the use of Search to AMIE in this way: *"Search was adequate when I had a good idea of what the differential ought to be to start with, but there were some cases where I was only able to generate 3 or 4 because I really wasn't sure. If I put in 'infectious causes of headache' [to the search engine] those were not very helpful."*, whereas *"[AMIE] was required to pull some additional diagnoses that may not have been the*

*We note that these comments are from a model trained in late 2023.

final diagnosis but would be important to think about.".

Need for Clinical Judgment and Critical Thinking.

While AMIE could expand the diagnostic considerations, clinicians consistently highlighted the model's limitations and the need to apply clinical judgment. They noted that AMIE could make mistakes, particularly in complex or atypical cases (C5) and could be influenced by the line of questioning, which could be misleading for individuals without medical expertise (C1, C3). This point is consistent with C2's view that AMIE was intuitive for them as a clinician but might be inaccurate for those without a clinical background.

The following quotes capture these themes in their own words: C1: *"I walked into it thinking I could ask what ever I want, but if it was something that could not be inferred from the case I might get a response that isn't real or appropriate.* C3: *"The biggest thing I think that I had a concern about was inaccuracy for someone who does not have a clinical background".* But the clinicians found ways to work around these limitations by leveraging their expertise or other resources to validate responses.

Variable Effectiveness Based on Case Complexity.

AMIE's helpfulness varied depending on the specifics of the case presentation. It was perceived as most useful for straightforward cases with clear pathognomonic signs, offering a quick and efficient way to confirm or expand upon the clinician's initial impressions (C1, C3). However, the model's limitations were more apparent in complex cases. Clinicians noted it could become distracted by individual symptoms and fail to synthesize the information holistically, which limited its usefulness in these scenarios (C1).

LLM as a Collaborative Learning Tool.

Clinicians viewed AMIE as a valuable learning resource, particularly for medical education, due to its ability to quickly provide a range of potential diagnoses and their explanations from a single source (C1, C3). They highlighted the potential for AMIE to 'upskill' clinical providers through this expanded perspective and the provision of detailed explanations (C1, C3). Clinician C2 expressed a desire to have this tool available every day as an aid for their clinical practice. Interface and User Experience: Clinicians found the conversational interface intuitive and easy to use (C2), highlighting that this enabled them to interact with AMIE efficiently without adding substantial time to the task. However, they indicated AMIE's helpfulness could be improved by providing more explicit guidance on how to interact with it effectively (C5) and by enabling the model to communicate uncertainty.

SI.6 Cases

Table SI.2 | NEJM CPC Case IDs. A full list of the NEJM CPC cases used in this study.

Case ID	Issue	Year	Title
Case ID	Issue	Year	Title
1215969	18	2013	A 32-Year-Old Woman with Recurrent Episodes of Altered Consciousness
1209273	19	2013	A 35-Year-Old Woman with Recurrent Goiter and Ductal Carcinoma
1302333	20	2013	A 29-Year-Old Man with Anemia and Jaundice
1302332	21	2013	A 68-Year-Old Man with Metastatic Melanoma
1209275	22	2013	A 51-Year-Old Woman with Epistaxis and Oral Mucosal Ulcers
1208154	23	2013	A 54-Year-Old Woman with Abdominal Pain, Vomiting, and Confusion
1201415	24	2013	A 53-Year-Old Woman with Erythroderma, Pruritus, and Lymphadenopathy
1209278	25	2013	A 71-Year-Old Man with Hematuria and a Mass in the Bladder
1208152	26	2013	A 46-Year-Old Woman with Muscle Pain and Swelling
1209277	27	2013	A 6.5-Month-Old Boy with Fever, Rash, and Cytopenias
1304164	28	2013	A 52-Year-Old Man with Cardiac Arrest after an Acute Myocardial Infarction
1305985	29	2013	A 32-Year-Old HIV-Positive African Man with Dyspnea and Skin Lesions
1214218	30	2013	A 19-Year-Old Man with Otalgia, Slurred Speech, and Ataxia
1304165	31	2013	A 29-Year-Old Man with Abdominal Pain, Fever, and Weight Loss
1208153	32	2013	A 55-Year-Old Woman with Autoimmune Hepatitis, Cirrhosis, Anorexia, and Abdominal Pain
1215968	33	2013	A 40-Year-Old Woman with Abdominal Pain, Weight Loss, and Anxiety about Cancer
1302431	34	2013	A 69-Year-Old Man with Dizziness and Vomiting
1209274	35	2013	A 77-Year-Old Man with Confusion and Malaise

Continued on next page...

Table SI.2 – continued from previous page

Case ID	Issue	Year	Title
1209651	37	2013	A 41-Year-Old Woman with Malaise and Chest and Abdominal Pain
1310002	38	2013	A 30-Year-Old Man with Fever and Lymphadenopathy
1215967	39	2013	A 57-Year-Old Woman with Painful Bullous Skin Lesions
1304051	40	2013	A 36-Year-Old Man with Agitation and Paranoia
1214217	1	2014	A 32-Year-Old Man with Loss of Vision and a Rash
1214220	3	2014	A 61-Year-Old Woman with Gastrointestinal Symptoms, Anemia, and Acute Kidney Injury
1305990	4	2014	A 39-Year-Old Man with Night Sweats and Abdominal Pain
1310004	5	2014	A 59-Year-Old Man with Fever, Confusion, Thrombocytopenia, Rash, and Renal Failure
1208155	6	2014	A 35-Day-Old Boy with Fever, Vomiting, Mottled Skin, and Severe Anemia
1302331	7	2014	A 27-Year-Old Man with Diarrhea, Fatigue, and Eosinophilia
1305992	9	2014	A 34-Year-Old Woman with Increasing Dyspnea
1304162	10	2014	A 45-Year-Old Man with a Rash
1314242	12	2014	A 59-Year-Old Man with Fatigue, Abdominal Pain, Anemia, and Abnormal Liver Function
1305994	13	2014	A 41-Year-Old Man with Fever and Abdominal Pain after Stem-Cell Transplantation
1305987	14	2014	An 11-Month-Old Girl with Developmental Delay
1400839	16	2014	A 46-Year-Old Woman in Botswana with Postcoital Bleeding
1304161	18	2014	A 32-Year-Old Man with a Rash, Myalgia, and Weakness
1400841	20	2014	A 65-Year-Old Man with Dyspnea and Progressively Worsening Lung Disease
1304163	22	2014	A 40-Year-Old Woman with Postpartum Dyspnea and Hypoxemia
1404140	23	2014	A 41-Year-Old Man with Fevers, Rash, Pancytopenia, and Abnormal Liver Function
1404139	24	2014	A 27-Year-Old Man with Severe Osteoporosis and Multiple Bone Fractures
1400842	25	2014	A 37-Year-Old Man with Ulcerative Colitis and Bloody Diarrhea
1400834	26	2014	A 21-Month-Old Boy with Lethargy, Respiratory Distress, and Abdominal Distention
1400833	27	2014	A 10-Month-Old Boy with Microcephaly and Episodic Cyanosis
1405886	28	2014	A 39-Year-Old Man with a Rash, Headache, Fever, Nausea, and Photophobia
1403307	29	2014	A 60-Year-Old Woman with Syncope
1405218	30	2014	A 29-Year-Old Man with Diarrhea, Nausea, and Weight Loss
1404517	31	2014	A 50-Year-Old Man with Back Pain, Fatigue, Weight Loss, and Knee Swelling
1406191	32	2014	A 78-Year-Old Woman with Chronic Sore Throat and a Tonsillar Mass
1310000	33	2014	A 60-Year-Old Man with Bone Pain report.
1305993	34	2014	A 7-Year-Old Boy with Focal Seizures and Progressive Weakness the literature.
1407131	35	2014	A 31-Year-Old Woman with Fevers, Chest Pain, and a History of HCV Infection
1310001	36	2014	An 18-Year-Old Woman with Fever, Pharyngitis, and Double Vision
1305989	37	2014	A 35-Year-Old Woman with Suspected Mite Infestation
1410935	38	2014	An 87-Year-Old Man with Sore Throat, Hoarseness, Fatigue, and Dyspnea
1410938	39	2014	A 9-Year-Old Girl with Crohn's Disease and Pulmonary Nodules
1404518	40	2014	A 57-Year-Old Man with Inguinal Pain, Lymphadenopathy, and HIV Infection
1408601	1	2015	A 66-Year-Old Woman with Metastatic Breast Cancer after Endocrine Therapy
1410939	2	2015	A 25-Year-Old Man with Abdominal Pain, Syncope, and Hypotension
1410936	3	2015	A 60-Year-Old Woman with Abdominal Pain, Dyspnea, and Diplopia
1410940	4	2015	A 49-Year-Old Man with Obtundation Followed by Agitation and Acidosis
1314241	5	2015	A 69-Year-Old Woman with Recurrent Skin Lesions after Treatment for Lymphoma
1411928	6	2015	A 16-Year-Old Boy with Coughing Spells
1409840	8	2015	A 68-Year-Old Man with Multiple Myeloma, Skin Tightness, Arthralgias, and Edema
1409839	9	2015	A 31-Year-Old Man with Personality Changes and Progressive Neurologic Decline
1314239	10	2015	A 15-Year-Old Girl with Graves' Disease and Psychotic Symptoms
1415165	11	2015	A 28-Year-Old Woman with Headache, Fever, and a Rash
1400837	12	2015	A Newborn Boy with Respiratory Distress, Lethargy, and Hypernatremia
1415172	13	2015	A 27-Year-Old Woman with Arthralgias and a Rash
1415200	14	2015	A 58-Year-Old Woman with Shortness of Breath
1501149	16	2015	A 9-Year-Old Girl with Loss of Consciousness and Seizures
1415170	18	2015	A 41-Year-Old Woman with Decreased Vision in the Left Eye and Diplopia
1415757	19	2015	A 71-Year-Old Man with Chest Pain and Shortness of Breath
1404334	20	2015	A Newborn Girl with Hypotension, Coagulopathy, Anemia, and Hyperbilirubinemia
1411439	21	2015	A 37-Year-Old American Man Living in Vietnam, with Fever and Bacteremia
1501310	22	2015	A 20-Year-Old Man with Sore Throat, Fever, Myalgias, and a Pericardial
1406415	23	2015	A 51-Year-Old Woman with Headache, Cognitive Impairment, and Weakness
1501763	24	2015	A 28-Year-Old Pregnant Woman with Fever, Chills, Headache, and Fatigue
1400836	25	2015	An 8-Year-Old Girl with a Chest-Wall Mass and a Pleural Effusion

Continued on next page...

Table SI.2 – continued from previous page

Case ID	Issue	Year	Title
1400843	26	2015	A 9-Month-Old Girl with Recurrent Fevers
1310003	27	2015	A 78-Year-Old Man with Hypercalcemia and Renal Failure
1404335	29	2015	A 38-Year-Old Pregnant Woman with Headache and Visual Symptoms
1415169	30	2015	A 50-Year-Old Man with Cardiogenic Shock
1406663	31	2015	A 29-Year-Old Man with Thymoma, Diarrhea, and Weight Loss
1503830	32	2015	A 57-Year-Old Man with Severe Pneumonia and Hypoxemic Respiratory Failure
1413304	33	2015	A 57-Year-Old Woman with Hair Loss and Deepening Voice
1502151	34	2015	A 36-Year-Old Woman with a Lung Mass, Pleural Effusion, and Hip Pain
1505527	35	2015	A 72-Year-Old Woman with Proteinuria and a Kidney Mass
1410941	36	2015	A 27-Year-Old Woman with a Lesion of the Ear Canal
1504839	37	2015	A 76-Year-Old Man with Fevers, Leukopenia, and Pulmonary Infiltrates
1507212	39	2015	A 22-Year-Old Man with Hypoxemia and Shock
1405204	40	2015	A 40-Year-Old Homeless Woman with Headache, Hypertension, and Psychosis
1408595	41	2015	A 14-Year-Old Boy with Immune and Liver Abnormalities
1501306	1	2016	An 18-Year-Old Man with Fever, Abdominal Pain, and Thrombocytopenia
1502149	2	2016	An 84-Year-Old Woman with Chest Pain, Dyspnea, and a Rash
1413305	3	2016	A 9-Year-Old Girl with Intermittent Abdominal Pain
1508550	4	2016	A 58-Year-Old Woman with a Skin Ulcer, Fever, and Lymphadenopathy
1509361	5	2016	A 43-Year-Old Man with Altered Mental Status and a History of Alcohol Use
1408597	6	2016	A 10-Year-Old Boy with Abdominal Cramping and Fevers
1509455	7	2016	An 80-Year-Old Man with Weight Loss, Abdominal Pain, Diarrhea, and an Ileocecal Mass
1505680	8	2016	A 71-Year-Old Man with Recurrent Fevers, Hypoxemia, and Lung Infiltrates
1512452	9	2016	A 29-Year-Old Man with Dyspnea and Chest Pain
1501148	10	2016	A 22-Year-Old Man with Sickle Cell Disease, Headache, and Difficulty Speaking
1512458	11	2016	A 12-Year-Old Boy with Malaise, Fevers, Abdominal Pain, and Pallor
1503831	12	2016	An 8-Year-Old Boy with an Enlarging Mass in the Right Breast
1514473	14	2016	A 37-Year-Old Woman with Adult-Onset Psychosis
1516449	15	2016	A 32-Year-Old Man with Olfactory Hallucinations and Paresthesias
1516451	16	2016	A 31-Year-Old Pregnant Woman with Fever
1516452	17	2016	A 60-Year-Old Woman with Increasing Dyspnea
1600612	18	2016	A 52-Year-Old Woman with a Pleural Effusion
1512457	19	2016	A 65-Year-Old Man with End-Stage Renal Disease and a Pruritic Rash
1600611	20	2016	A 50-Year-Old Man with Cloudy Vision, Hearing Loss, and Unsteadiness
1600838	21	2016	A 32-Year-Old Man in an Unresponsive State
1601838	22	2016	A 65-Year-Old Man with Syncope, Dyspnea, and Leg Edema
1601840	23	2016	A 46-Year-Old Man with Somnolence after Orthopedic Surgery
1503829	24	2016	A 66-Year-Old Man with Malaise, Weakness, and Hypercalcemia
1602815	25	2016	A 33-Year-Old Man with Rectal Pain and Bleeding
1607091	27	2016	A 71-Year-Old Woman with Müllerian Carcinoma, Fever, Fatigue, and Myalgias
1609109	29	2016	A 53-Year-Old Woman with Pain and a Mass in the Breast
1609309	30	2016	A 63-Year-Old Woman with Bipolar Disorder, Cancer, and Worsening Depression
1610097	31	2016	A 53-Year-Old Man with Diplopia, Polydipsia, and Polyuria
1610098	32	2016	A 20-Year-Old Man with Gynecomastia
1609308	33	2016	A 30-Year-Old Woman with Severe Lower Abdominal Pain and Chills
1610096	34	2016	A 17-Year-Old Boy with Myopia and Craniofacial and Skeletal Abnormalities
1610099	36	2016	A 50-Year-Old Man with Acute Liver Injury
1509539	37	2016	An 86-Year-Old Woman with Leukocytosis and Splenomegaly
1610101	38	2016	A 52-Year-Old Woman with Recurrent Oligodendroglioma
1613468	40	2016	A 14-Month-Old Girl with Recurrent Vomiting
1613459	1	2017	A 70-Year-Old Woman with Gradually Progressive Loss of Language
1613467	2	2017	An 18-Year-Old Woman with Acute Liver Failure
1610713	3	2017	A 62-Year-Old Man with Cardiac Sarcoidosis and New Diplopia and Weakness
1613465	4	2017	A 2-Month-Old Girl with Growth Retardation and Respiratory Failure
1610100	5	2017	A 19-Year-Old Man with Hematuria and a Retroperitoneal Mass
1613461	6	2017	A 57-Year-Old Woman with Fatigue, Sweats, Weight Loss, Headache, and Skin Lesions
1613462	7	2017	A 73-Year-Old Man with Confusion and Recurrent Epistaxis
1616024	9	2017	A 27-Year-Old Woman with Nausea, Vomiting, Confusion, and Hyponatremia
1616020	10	2017	A 6-Month-Old Boy with Gastrointestinal Bleeding and Abdominal Pain
1616023	11	2017	A 61-Year-Old Woman with Leg Swelling, Back Pain, and Hydronephrosis

Continued on next page...

Table SI.2 – continued from previous page

Case ID	Issue	Year	Title
1616395	12	2017	A 34-Year-Old Man with Nephropathy
1616022	13	2017	A 41-Year-Old Man with Hearing Loss, Seizures, Weakness, and Cognitive Decline
1616397	14	2017	A 20-Year-Old Man with Pain and Swelling of the Left Calf and a Purpuric Rash
1616396	15	2017	A 27-Year-Old Woman with Anemia, Thrombocytosis, and Skin Lesions after Travel Abroad
1616398	16	2017	A 69-Year-Old Woman with Urinary Incontinence
1616019	17	2017	A 14-Year-Old Boy with Acute Fear of Choking while Swallowing
1616394	18	2017	An 11-Year-Old Girl with Difficulty Eating after a Choking Incident
1701762	19	2017	A 53-Year-Old Woman with Leg Numbness and Weakness
1616401	20	2017	A 48-Year-Old Man with Weight Loss, Confusion, Skin Lesions, and Pancytopenia
1616399	22	2017	A 21-Year-Old Woman with Fever, Headache, and Myalgias
1703512	23	2017	A 9-Day-Old Girl with Vomiting, Acidosis, and Azotemia
1616393	24	2017	An 8-Month-Old Girl with Fever and an Abdominal Mass
1706099	25	2017	A 45-Year-Old Man with Headache, Fever, and Lymphadenopathy
1616402	26	2017	A 63-Year-Old Woman with Fever, Hypotension, and Hypoxemia
1706108	29	2017	A 59-Year-Old Woman with Pain and Swelling in the Right Hand and Ankles
1706100	30	2017	A 65-Year-Old Woman with Altered Mental Status, Bacteremia, and Acute Liver Failure
1706106	31	2017	A 19-Month-Old Girl with Failure to Thrive
1703513	32	2017	A 64-Year-Old Man with Dyspnea, Wheezing, Headache, Cough, and Night Sweats
1707557	34	2017	A 76-Year-Old Man with Fever, Weight Loss, and Weakness
1710564	35	2017	A 57-Year-Old Woman with Hypoesthesia and Weakness in the Legs and Arms
1710565	36	2017	A 30-Year-Old Man with Fatigue, Rash, Anemia, and Thrombocytopenia
1710563	37	2017	A 36-Year-Old Man with Unintentional Opioid Overdose
1706109	38	2017	A 20-Year-Old Woman with Seizures and Progressive Dystonia adolescence.
1707558	39	2017	A 41-Year-Old Woman with Recurrent Chest Pain
1710566	40	2017	A 32-Year-Old Woman with Headache, Abdominal Pain, Anemia, and Thrombocytopenia
1712222	1	2018	A 39-Year-Old Woman with Rapidly Progressive Respiratory Failure
1701763	2	2018	A 41-Year-Old Woman with Vision Disturbances and Headache
1706107	3	2018	A 5-Month-Old Boy with Hypoglycemia
1706110	4	2018	A Newborn with Thrombocytopenia, Cataracts, and Hepatosplenomegaly
1707556	5	2018	A 63-Year-Old Man with Confusion after Stem-Cell Transplantation
1712223	6	2018	A 35-Year-Old Woman with Headache, Subjective Fever, and Anemia
1712226	7	2018	A 25-Year-Old Man with New-Onset Seizures
1712225	8	2018	A 55-Year-Old Woman with Shock and Labile Blood Pressure costeroids.
1800321	9	2018	A 55-Year-Old Man with HIV Infection and a Mass on the Right Side of the Face
1712224	10	2018	An 84-Year-Old Man with Painless Unilateral Testicular Swelling
1800323	11	2018	A 48-Year-Old Woman with Recurrent Venous Thromboembolism
1800322	12	2018	A 30-Year-Old Woman with Cardiac Arrest
1800333	13	2018	A 53-Year-Old Man with Cardiomyopathy and Recurrent Ventricular Tachycardia
1800337	14	2018	A 68-Year-Old Woman with a Rash, Hyponatremia, and Uveitis
1800339	15	2018	An 83-Year-Old Woman with Nausea, Vomiting, and Confusion
1712227	16	2018	A 45-Year-Old Man with Fever, Thrombocytopenia, and Elevated Aminotransferase Levels
1916254	13	2020	A 29-Year-Old Man with High Blood Pressure, Renal Insufficiency,
1909623	14	2020	A 37-Year-Old Man with Joint Pain and Eye Redness
1913477	15	2020	A 79-Year-Old Man with Hyponatremia and Involuntary Movements of the Arm and Face
1916258	16	2020	A 47-Year-Old Woman with Recurrent Melanoma and Pulmonary Nodules
2002418	17	2020	A 68-Year-Old Man with Covid-19 and Acute Kidney Injury
2002417	18	2020	A 73-Year-Old Man with Hypoxemic Respiratory Failure and Cardiac Dysfunction
2004975	24	2020	A 44-Year-Old Woman with Chest Pain, Dyspnea, and Shock
2004977	25	2020	A 47-Year-Old Woman with a Lung Mass
2004976	26	2020	A 60-Year-Old Woman with Altered Mental Status and Weakness on the Left Side lesions.
1913472	27	2020	A 53-Year-Old Woman with Headache and Gait Imbalance
2004981	28	2020	A 64-Year-Old Man with Intrusive Thoughts and Fear of Being Poisoned
2002420	30	2020	A 54-Year-Old Man with Sudden Cardiac Arrest
1904039	31	2020	A 48-Year-Old Man with Lymphoma and Abdominal Pain
2004996	32	2020	A 63-Year-Old Man with Confusion, Fatigue, and Garbled Speech
1916257	33	2020	A 55-Year-Old Man with Abdominal Pain, Joint Swelling and Skin Legions
2002415	34	2020	A 74-Year-Old Man with Chronic Kidney Disease
2002412	35	2020	A 59-Year-Old Woman with Type 1 Diabetes Mellitus and Obtundation
2027077	36	2020	A 72-Year-Old Woman with Dark Urine and Weakness

Continued on next page...

Table SI.2 – continued from previous page

Case ID	Issue	Year	Title
2004979	37	2020	A 35-Year-Old Man with Lymphadenopathy and Petechiae
2004991	38	2020	A 52-Year-Old Man with Cancer and Acute Hypoxemia and ARDS
2027078	39	2020	A 29-Month-Old Boy with Seizure and Hypocalcemia
2027083	40	2020	A 24-Year-Old Man with Headache and Covid-19
1916251	41	2020	A 62-Year-Old Man with Memory Loss and Odd Behavior
2027084	1	2021	A 76-Year-Old Woman with Lethargy and Altered Mental Status
2027086	2	2021	A 26-Year-Old Pregnant Woman with Ventricular Tachycardia and Shock
2002416	3	2021	A 48-Year-Old Man with Transient Vision Loss
2027088	4	2021	A 70-Year-Old Woman with Dyspnea on Exertion and Abnormal Findings on Chest Imaging
1913474	5	2021	A 68-Year-Old Man with Delirium and Renal Insufficiency
2027089	6	2021	A 65-Year-Old Man with Eye Pain and Decreased Vision
2027093	7	2021	A 19-Year-Old Man with Shock, Multiple Organ Failure, and Rash
2027094	9	2021	A 16-Year-Old Boy with Headache, Abdominal Pain, and Hypertension
2027090	10	2021	A 70-Year-Old Man with Depressed Mood, Unsteady Gait, and Urinary Incontinence
2100273	11	2021	A 39-Year-Old Woman with Fever, Flank Pain, and Inguinal Lymphadenopathy
2100276	12	2021	A 78-Year-Old Man with a Rash on the Scalp and Face
2100277	13	2021	A Newborn Girl with a Neck Mass
2100275	14	2021	A 64-Year-Old Woman with Fever and Pancytopenia
2100274	15	2021	A 76-Year-Old Woman with Nausea, Diarrhea, and Acute Kidney Failure
2100278	16	2021	A 37-Year-Old Woman with Abdominal Pain and Aortic Dilatation
2100281	17	2021	An 82-Year-Old Woman with Pain, Swelling, and Ecchymosis of the Left Arm
2100283	18	2021	An 81-Year-Old Man with Cough, Fever, and Shortness of Breath
2100272	19	2021	A 54-Year-Old Man with Irritability, Confusion, and Odd Behaviors
2004992	20	2021	A 69-Year-Old Man with Ataxia
2107344	21	2021	A 33-Year-Old Pregnant Woman with Fever, Abdominal Pain, and Headache
2103460	22	2021	A 64-Year-Old Woman with Cognitive Impairment, Headache, and Memory Loss
2103461	23	2021	A 41-Year-Old Woman with Bloody Stools and Thrombocytopenia
2107345	24	2021	A 63-Year-Old Woman with Fever, Sore Throat, and Confusion
2100282	25	2021	A 48-Year-Old Man with Fatigue and Leg Swelling
2107350	28	2021	A 37-Year-Old Woman with Covid-19 and Suicidal Ideation history.
2107346	29	2021	A 12-Month-Old Boy with Fever and Developmental Regression
2107347	30	2021	A 47-Year-Old Man with Recurrent Unilateral Head and Neck Pain
2027096	31	2021	A 21-Year-Old Man with Sore Throat, Epistaxis, and Oropharyngeal Petechiae
2107351	32	2021	A 14-Year-Old Girl with Swelling of the Jaw and Hypercalcemia
2107352	33	2021	A 68-Year-Old Man with Painful Mouth Ulcers
2027080	34	2021	A 38-Year-Old Man with Altered Mental Status and New Onset of Seizures
2107356	35	2021	A 50-Year-Old Woman with Pain in the Left Upper Quadrant and Hypoxemia
2107357	36	2021	A 22-Year-Old Man with Pain and Erythema of the Left Hand
2107353	37	2021	A 60-Year-Old Man with Fevers, Fatigue, Arthralgias, a Mouth Ulcer, and a Rash
2107354	38	2021	A 76-Year-Old Woman with Abdominal Pain, Weight Loss, and Memory Impairment
2107355	39	2021	A 26-Year-Old Woman with Respiratory Failure and Altered Mental Status
2107358	40	2021	A 9-Year-Old Boy with Transient Weakness, Facial Droop, and Slurred Speech
2115844	1	2022	A 67-Year-Old Man with Motor Neuron Disease and Odd Behaviors during Sleep
2115847	2	2022	A 70-Year-Old Man with a Recurrent Left Pleural Effusion
2115850	3	2022	A 14-Year-Old Boy with Fever, Joint Pain, and Abdominal Cramping
2107349	4	2022	A 55-Year-Old Man with Bilateral Hearing Loss and Eye Redness
2115852	5	2022	A 65-Year-Old Woman with Rapidly Progressive Weakness in the Right Arm
2115848	6	2022	A 68-Year-Old Man with Fatigue, Weight Loss, and Hyperglycemia
2115853	7	2022	A 65-Year-Old Woman with Depression, Recurrent Falls, and Inability to Care for Herself
2027087	8	2022	A 54-Year-Old Woman with Episodes of Swelling
2115846	9	2022	A 56-Year-Old Woman with Fever, Myalgias, Diarrhea, and Cough
2201230	10	2022	A 78-Year-Old Man with Marked Ventricular Wall Thickening
2201232	11	2022	An 80-Year-Old Woman with Pancytopenia
2115855	12	2022	A 41-Year-Old Woman with Transient Ischemic Attack and Mitral Valve Masses
2201233	13	2022	A 56-Year-Old Man with Myalgias, Fever, and Bradycardia
2115856	14	2022	A 57-Year-Old Man with Chylous Ascites
2201234	15	2022	A 57-Year-Old Man with Persistent Cough and Pulmonary Opacities
2115849	16	2022	A 55-Year-Old Man with Fevers, Night Sweats, and a Mediastinal Mass
1909620	17	2022	A 17-Year-Old Boy with Chest Pain

Continued on next page...

Table SI.2 – continued from previous page

Case ID	Issue	Year	Title
2107348	18	2022	A 29-Year-Old Woman with Recurrent Fractures
2201231	19	2022	A 29-Year-Old Woman with Jaundice and Chronic Diarrhea
2115854	20	2022	A 25-Year-Old Man with Vision Changes
2115851	21	2022	A 17-Year-Old Girl with Fever and Cough
2201238	22	2022	A 34-Year-Old Woman with Cavitary Lung Lesions
2115858	23	2022	A 49-Year-Old Man with Hypoglycemia
2201244	24	2022	A 31-Year-Old Man with Perianal and Penile Ulcers, Rectal Pain, and Rash
2201241	25	2022	A 25-Year-Old Woman with Headache and Blurred Vision
2201247	26	2022	A 48-Year-Old Woman with Cystic Lung Disease metastasis.
2201246	27	2022	A 32-Year-Old Man with Confusion, Headache, and Fever
2100271	28	2022	A 59-Year-Old Man with Headache and Progressive Neurologic Dysfunction
2201236	29	2022	A 33-Year-Old Man with Chronic Diarrhea and Autoimmune Enteropathy
2201243	30	2022	A Newborn Girl with Hypoglycemia
2201239	31	2022	A 72-Year-Old Man with Heartburn, Nausea, and Inability to Eat
2201245	32	2022	A 76-Year-Old Man with Postoperative Cardiogenic Shock and Diffuse Rash
2201235	33	2022	An 11-Year-Old Girl with Redness of the Eyes paradigms.
2115857	34	2022	A 57-Year-Old Woman with Covid-19 and Delusions
2211355	36	2022	A 30-Year-Old Woman with Decreased Vision and Headache
2211357	37	2022	A 55-Year-Old Man with Fatigue, Weight Loss, and Pulmonary Nodules Plant Recipient
2201250	38	2022	A 21-Year-Old Woman with Fatigue and Weight Gain
2211363	39	2022	A 31-Year-Old Woman with Postpartum Abdominal Pain and Fever
2211360	40	2022	A 38-Year-Old Man with Exertional Chest Discomfort
2211366	1	2023	A 49-Year-Old Man with Hypokalemia and Paranoia
2201240	2	2023	A 76-Year-Old Man with Dizziness and Altered Mental Status
2211367	3	2023	A 16-Year-Old Girl with Abdominal Pain and Bloody Diarrhea
2201249	4	2023	A 56-Year-Old Man with Abnormal Results on Liver Testing
2211364	5	2023	A 67-Year-Old Man with Interstitial Lung Disease, Fever, and Myalgias
2211368	6	2023	A 68-Year-Old Man with Recurrent Strokes
2211369	7	2023	A 70-Year-Old Man with Covid-19, Respiratory Failure, and Rashes
2211370	8	2023	A 71-Year-Old Woman with Refractory Hemolytic Anemia
2211356	9	2023	A 20-Year-Old Man with Shortness of Breath and Proteinuria
2211365	10	2023	A 27-Year-Old Man with Convulsions
2211516	11	2023	A 67-Year-Old Man with Mantle-Cell Lymphoma and Hypoxemia
2211375	12	2023	A 44-Year-Old Woman with Muscle Weakness and Myalgia
2300896	13	2023	A 25-Year-Old Woman with Abdominal Pain and Jerking Movements
2211511	14	2023	A 31-Year-Old Man with Redness of the Right Eye
2300895	15	2023	A 33-Year-Old Man with Paresthesia of the Arms and Legs
2201237	16	2023	A 13-Year-Old Boy with Depression and Hypotension
2201248	17	2023	A 58-Year-Old Woman with Fatigue, Abdominal Bloating, and Eosinophilia
2300894	18	2023	A 19-Year-Old Woman with Dyspnea and Tachypnea
2211512	19	2023	An 80-Year-Old Man with Left Foot Drop
2300899	20	2023	A 52-Year-Old Man with a Solitary Fibrous Tumor and Hypoglycemia
2300904	21	2023	A 61-Year-Old Man with Eyelid Swelling
2300898	22	2023	A 59-Year-Old Woman with Hypotension and Electrocardiographic Changes
2300906	23	2023	A 21-Year-Old Man with Progressive Dyspnea
2300911	24	2023	A 43-Year-Old Man with a Pulmonary Nodule
2211358	25	2023	An 18-Year-Old Man with Fever and Foot Pain
2211422	26	2023	A 15-Year-Old Girl with Abdominal Pain and an Ovarian Mass
